# Effect of TMS laterality on clinical outcomes in treatment resistant depression patients with comorbid anxiety - a retrospective study

**DOI:** 10.3389/fpsyt.2025.1494811

**Published:** 2025-03-27

**Authors:** Thomas Caussat, Brian Blair, Lindsay M. Oberman

**Affiliations:** ^1^ Charles E. Schmidt College of Medicine, Florida Atlantic University, Boca Raton, FL, United States; ^2^ Health Morsani College of Medicine, University of South Florida, Tampa, FL, United States; ^3^ National Institute of Mental Health, National Institute of Health (NIH), Bethesda, MD, United States

**Keywords:** repetitive transcranial magnetic stimulation, anxious depression, dorsolateral prefrontal cortex, unilateral, bilateral

## Abstract

**Objectives:**

High-frequency repetitive transcranial magnetic stimulation (rTMS) of the left-hemisphere dorsolateral prefrontal cortex (DLPFC) is FDA cleared for the treatment of adult treatment-resistant major depressive disorder (MDD). Though off-label, sequential bilateral stimulation (SBS), which combines high-frequency left-hemisphere and low-frequency right-hemisphere DLPFC stimulation, is offered in various clinics to treat depression with comorbid anxiety. Few systematic studies investigate the comparative efficacy of the SBS protocol versus the FDA-label protocol for the clinical management of depression with comorbid anxiety. The objective of the current study was to compare the efficacy of HF-LUS to that of SBS within a clinical setting where both are offered to patients with anxious depression. Based on both theories of the pathophysiology of anxious depression as well as clinical practice, we hypothesized that SBS would result in greater symptom reduction as compared to HF-LUS.

**Methods:**

This open label, retrospective cohort study included 86 patients with MDD and comorbid anxiety who received either high frequency left unilateral stimulation (HF-LUS) (n=44) or SBS (n=42). Patient Health Questionnaire 9 (PHQ9), General Anxiety Disorder 7 (GAD7) questionnaire, a self-reported depression (SRD) Likert scale, and a self-reported anxiety (SRA) Likert scale were used to quantify changes in depressive and anxiety symptoms.

**Results:**

Inconsistent with our hypothesis, both groups saw a significant improvement in depression and anxiety symptoms with no difference in course nor degree of improvement. Improvements in depression and anxiety were significantly positively correlated in both bilateral and unilateral cohorts.

**Conclusions:**

Bilateral rTMS may not provide any additional therapeutic advantages over the standard FDA-cleared left unilateral rTMS protocol for anxious depressive patients.

## Introduction

Transcranial magnetic stimulation (TMS) is a non-invasive neuromodulation technology that applies fluctuating magnetic fields over the scalp and generates targeted electrical currents in the brain, leading to neuronal depolarization (1). The non-invasive nature of this modality along with its rare occurrence of side effects (2) has since rendered it an attractive tool in both research and clinical domains. As repetitive application of TMS (rTMS) has plastic effects on the brain with clinically meaningful durability (3, 4), it has also gained popularity as a treatment in the emerging field of interventional psychiatry.

Following several large-scale clinical trials supporting the antidepressant efficacy and safety of rTMS (5, 6), the FDA cleared high frequency (HF - 10Hz) stimulation of the left dorsolateral prefrontal cortex (DLPFC) for the treatment of adult treatment-resistant major depressive disorder (MDD) in 2008 and more recently the same protocol was cleared for reduction of comorbid anxiety symptoms in adult patients with depression, otherwise known as anxious depression, in 2022. In addition to MDD, studies indicate the potential efficacy of rTMS in treating a number of other psychiatric disorders, such as posttraumatic stress disorder (7), obsessive compulsive disorder (8), bipolar disorder (9, 10), and anxiety disorders (7, 11, 12). Anxiety disorders frequently co-occur with major depressive disorder (MDD), with a substantial proportion of individuals with MDD also experiencing significant anxiety symptoms (13). This comorbidity has been associated with poorer treatment response across multiple modalities, including pharmacotherapy and psychotherapy (14). However, the impact of rTMS on this subgroup remains an area of active investigation, with limited data directly comparing different stimulation protocols for anxious depression. In this study, we focus specifically on patients with comorbid anxious depression treated either with the standard unilateral protocol or the bilateral protocol. All patients in the study endorsed both depressive and anxiety symptoms that significantly impaired their quality of life.

### Hemispheric lateralization

Electroencephalography (EEG) recordings have shown that negative mood and depression are associated with relatively greater activity in the right hemisphere’s (RH) frontal cortex as compared to the analogous region in the left hemisphere (LH) (15, 16). Consistent with this, neuroimaging studies report that in uni-polar depressed patients the LH is characterized by hypometabolism and by hypermetabolism in the RH (17, 18). Studies also find that the severity of depression correlates positively with RH hyperactivity (17, 19). Studies on unilateral brain lesions, which offer an opportunity to study hemispheric balance with one healthy hemisphere operating predominantly without contra hemispheric influence, find that tumors and ischemia in the left hemisphere are frequently accompanied by depressed mood, while similar lesions in the right hemisphere cause euphoria (20–22). Also noteworthy is that the frequency and severity of post-stroke depression is higher in patients with left hemispheric lesions compared with right hemispheric patients (23–25). Within the same vein, inactivation of the left hemisphere via sedative injection into the left carotid artery (effectively isolating the RH), produces crying, pessimistic statements, guilt, complaints, and worries about the future, whereas sedation of the right hemisphere results in smiling, laughing, mimicry, euphoria, and lack of apprehension (26, 27).

The symptoms of anxious depression may be understood in the context of an imbalance in hemispheric activity. Pessimism, negative thinking patterns, unconstructive attribution style, as well as guilt and self-blame thoughts have all been associated with RH hyperactivity (28–31). Difficulties in initiating and maintaining a healthy sleep pattern may also be related to the RH hyperactivity when considering its role in maintaining alertness and vigilance (19) and its role in modulating physiological symptoms of anxiety, such as sweating and increased heart rate (32). Conversely, the relative hypoactivity of the LH may account for the lack of motivation and inability to experience pleasure – anhedonia, as well as the indecisiveness that is associated with depression, as these functions are primarily thought to be processed by the LH (19). Studies on unilateral brain lesions also find that tumors and ischemia in the left hemisphere are frequently accompanied by depressed mood, while similar lesions in the right hemisphere cause euphoria (20–22).

### rTMS parameters

Typically, high-frequency (∼10 Hz) rTMS is thought to increase local cortical activity, while low-frequency (∼1 Hz) rTMS is thought to result in local cortical suppression (33, 34). In accordance with this assumption, studies have found clinical improvements in depression when administering high-frequency left unilateral stimulation (HF-LUS) (5, 35–37), low frequency right unilateral stimulation (35), and Sequential Bilateral Stimulation (SBS), which combines high frequency, left DLPFC stimulation and low frequency, right DLPFC stimulation (36). While all three protocols result in symptom improvements compared to sham (placebo) controls, there is contradictory data in the literature leading to a need for head-to-head comparisons of various protocols superiority (38, 39). Even the most recently pooled data in systematic reviews and meta-analyses, including a review by Aaronson and colleagues (40) which collected data from 111 practice sites in 2022, concluded that there was no significant difference in efficacy between unilateral and bilateral protocols. While their study was retrospective, it provides valuable insight that aligns with our findings.

In spite of approximately half of patients with MDD seeking treatment in the clinic also endorsing significant anxiety (41), patients with comorbid anxiety disorders are often excluded from rTMS studies focused exclusively on MDD. Consequently, while a growing body of findings show promise in patients with anxiety disorders and anxiety symptoms comorbid to other psychiatric pathologies (11, 12, 42), few studies have investigated which treatment parameters best remediate comorbid anxiety symptoms in those with depression in a clinical setting. Our study aims to investigate whether the SBS protocol, which is commonly offered clinically to depressive patients with significant anxiety, provides significant clinical benefit over the FDA cleared HF-LUS. We hypothesized that, by tackling the inter-hemispheric imbalance from both sides simultaneously, SBS treatment may be more effective than unilateral stimulation for this subpopulation of patients.

## Methods

Patients who sought treatment signed a consent form to have their information utilized for research purposes as part of their intake. Patients, TMS technicians, and those analyzing the data were unblinded to treatment protocol. This study was determined to be exempt from IRB review under category # 4(ii), as detailed in 45 CFR 46.104(d) by BRANY IRB Services.

### Participants

Patients were assigned to a treatment protocol (cohort) based upon their qualitative report of symptoms obtained by clinic staff during intake. Patients who reported that depressive symptoms alone were the primary cause of impairment were assigned to the unilateral protocol, while those who endorsed both depressive and anxiety symptoms as equally debilitating were assigned to the bilateral protocol. Cohort assignment was not randomized but was based on these patient-reported symptoms during intake interviews. While quantitative symptom severity metrics were also collected as part of intake, these values were used as baseline values prior to treatment and not factored in cohort assignment. Inclusion criteria involved patients with longstanding treatment resistant depression with comorbid anxiety symptoms or anxiety disorder who underwent between 30 and 36 treatments of either unilateral or bilateral TMS stimulation. Patients were classified as having anxious depression if they had a GAD-7 score of at least 10 and a PHQ-9 score of at least 10. Patients were allowed to remain on psychotropic medication and psychotherapy regimens, but those receiving other treatments such as concurrent intranasal ketamine or other neuro-stimulatory treatments were excluded from this analysis. Data for this study was pooled from patients treated at the Neuro Wellness center for Depression in Coral Springs, FL between the years 2020-2022. The groups were not significantly different demographically or clinically and received comparable intensities of stimulation (p >0.05 for all categories) (**Table 1**).

**Table 1:** Population Demographics.

Characteritics	Categories	HF-LUS	SBS	Total
Demographics	N	44	42	86
	Male	13 (29.5%)	18 (42.9)	32 (37.2)
	Female	31 (70.5)	24 (57.1)	55 (62.8)
	Mean Age (s.d.)	53 (18.4)	47 (17.4)	50 (17.9)
	Min/Max Age	13/88	20/79	13/88
Treatment	* Mean Motor	L - 30.25	L - 29.5	L - 30.0
	Threshold (MT)		R - 34.3	R - 34.3
	Mean Treatment	L 36.3	L - 35.4	L - 36.0
	Intensity (1.2 x MT)		R - 41.2	R - 41.2
	Mean MT Change	L- 10.16%	L - 5.21%	L - 7.76%
	in Remapping		R - 1.6%	R 1.6%
Medications	SSRI	10 (22.7%)	9 (21.4%)	19 (22.1%)
	SNRI	10 (22.7)	6 (14.3)	16 (18.6)
	Atypical	10 (22.7)	7 (16.7)	17 (19.8)
	Antidepressants
	Seratonin	7 (16.0)	7 (16.7)	14 (16.3)
	Modulators
	Benzodiazepine	10 (22.7)	8 (19.0)	18 (20.9)
	Antipsychotic	8 (18.2)	6 (14.3)	14 (16.3)
	Mood Stabilizer	0	1 (2.4)	1 (1.2)
	Stimulants	2 (4.5)	5 (11.9)	7 (8.1)
	Anti-Convulsant	6 (13.6)	6 (14.3)	12 (14.0)
	Non-Benzo	1 (2.3)	4 (9.5)	5 (5.8)
	Anxiolytic
	Z Drug	0	2 (4.8)	2 (2.3)

Demographics of study subjects, treatment doses, and psychotropic medications taken during study period.

*Reported motor thresholds are an average between patient starting motor threshold and corrected motor threshold around week 3. Percent change of adjustment was not significantly different between the two cohorts.

### Measures

As part of the intake protocol, patients completed the Patient Health Questionnaire 9 (PHQ9) and General Anxiety Disorder 7 (GAD7) questionnaire, along with two self-report Likert scales of anxiety and depression symptom severity (i.e. the self-reported anxiety (SRA) scale and the self-reported depression (SRD) scale). The SRA and SRD scales are self-report Likert scales developed by our clinic to provide real-time assessments of patients’ subjective experiences of anxiety and depression symptoms throughout the treatment course. These scales range from 0 to 10, with higher scores indicating greater symptom severity. While not standardized or validated like the PHQ-9 and GAD-7, they offer practical utility in tracking symptom changes on a session-by-session basis, complementing the more comprehensive assessments. Patients also documented past and current medications at intake. During treatment, patients completed the SRA and SRD scales prior to every session and the PHQ9 and GAD7 at the end of every treatment week. Finally, patients completed the PHQ9, GAD7, SRA, and SRD as part of the discharge protocol once their treatment course had concluded.

The PHQ9 is a questionnaire utilized by clinicians as a screening and severity assessment tool for depression based upon the DSM-V diagnostic criteria for depressive disorders (43). The threshold score of ‘4’ or less (below 5) was used to define remission for our study, at or below which patients’ symptoms do not meet clinical criteria for mild depression/anxiety. The GAD7 is a questionnaire utilized by clinicians as a screening and severity assessment tool for anxiety disorders based upon the DSM-V diagnostic criteria for generalized anxiety disorder (44). Similar to the PHQ9, a score of ‘4’ or less was used to define remission for anxiety symptoms. Response was defined as a ≥ 50% improvement from baseline to post-treatment scores on the PHQ-9 and GAD-7. The SRA and SRD are Likert scales which assess a patient’s experience of anxiety and depression symptoms. The scales range from 0 to 10, 0 indicating no anxiety/depression and 10 indicating the worst and most debilitating anxiety/depression symptoms imaginable.

### Procedures

All patients received magnetic resonance imaging (MRI)-guided rTMS with the Nexstim Navigated Brain Stimulation (NBS) System 5. Prior to their first session, patients received a series of structural MRI scans including a T1-weighted MP-RAGE scan, a three-dimensional T1-weighted scan, and a gradient-echo scan.

On the first day of treatment, a trained TMS technician and the attending psychiatrist confirmed the relevant anatomical landmarks identified on the patient’s MRI by the interpreting radiologists (including the left- and right-hand knobs (in the primary motor cortex) and the left and right DLPFC). The individualized location of the M1 hand knobs are defined by anatomical criteria proposed by Ahdab and colleagues (45) and Yousry and colleagues (46) The NBS system employs an algorithm developed by Mylius and colleagues (47) to define the optimal DLPFC target locations. After these anatomical landmarks are identified and marked in the Nexstim interface program, the attending psychiatrist/privileged provider then determined the patient’s Motor Threshold (MT) and calculated treatment intensity prior to starting treatment.

### Motor mapping and MT estimation

Motor mapping was performed by the attending physician to determine the patient’s MT and contingent stimulation intensity according to standard procedures. MT is defined within the Nexstim manual as the minimum intensity that elicits an EMG motor evoked potential of 100-500 μV with a latency in the 12-25 ms range 50% of the time. Treatment intensity is then defined as 120% of the MT. Recent findings suggest that MT varies significantly across an rTMS treatment course (48). Thus, MT is reevaluated around week 3 (between treatments 10-15) for all patients to account for any changes in neuronal excitability and to ensure that stimulation target(s) are optimal. While both cohorts receive motor mapping of the left hemisphere, the SBS group additionally undergoes the same process for the right hemisphere. Once a patient’s MRI is uploaded, MT(s) are determined, and cortical targets are all tagged, technicians calibrate these targets with landmarks on patient’s head to begin MRI guided rTMS.

All patients received the FDA cleared treatment for depression that takes roughly 19 minutes and delivers 3000 pulses in total at a frequency of 10 HZ to the LH DLPFC. These pulses are spaced out in 75 trains, each lasting 4 seconds, delivering 40 pulses each, and spaced out by an 11 second intertrain interval. Once the left side protocol is complete, patients in the bilateral cohort are recalibrated in the machine for right-sided DLPFC stimulation. The right-sided protocol lasts 20 minutes and delivers 1200 pulses at 1 HZ in one single train spaced out by a 1 second interval. Right-sided stimulation was delivered at 120% of the Motor Threshold (MT), consistent with the stimulation parameters for the left-sided treatment.

The average participant received 36 treatments, allotted as 5 times a week for the first 30 sessions, and tapered off to 3 times a week for the final 6.

### Data analysis

Data was analyzed using SPSS version 26, with the exception of Fisher’s r to z transformation, which was performed using an online calculator (49) as transformation is not available on SPSS 26. A handful of patients went on to receive over thirty-six sessions, but only data up to treatment thirty-six was considered. This was done to standardize the treatment timeline.

Our primary outcome measure was the effect of protocol on improvements in anxiety, thus we used a factorial (2x2) ANOVA to determine if patients reported greater or lesser improvement when comparing their initial quantitative measurement of symptoms to their final value post treatment. We then derived Pearson’s correlation coefficients with net treatment proportional improvements ((Intake Score – Final score)/Intake score) by comparing SRA against SRD scores and GAD7 against PHQ9 in a two tailed analysis. Correlation coefficients were then compared via Fisher’s r to z transformation. We subsequently calculated patient response (≥ 50% improvement) and remission rates (final scores below 5, for both PHQ9 & GAD7) for all cohorts using PHQ9 and GAD7 and analyzed the means via chi-square. Finally, we used an ANCOVA to determine if treatment trajectories differed between protocols. Due to inconsistent reporting, several patients were missing mid-treatment GAD7 and PHQ9 entries. In order to replace this data without compromising the accuracy of the ANCOVA, we replaced missing data points by using the mean of nearby points in patients with 3 or fewer missing entries and excluded patients with more than 3 missing entries. As a result, 7 participants were excluded for PHQ9 analysis, leaving us with n=79 (unilateral n = 42, bilateral n = 37) and excluded 9 from the GAD7 analysis, leaving us with n=77 (unilateral n= 41, bilateral n= 36).

## Results

All participants tolerated the TMS treatment without any adverse medical events.

### Metrics of depression – PHQ9 & SRD

**Figure 1:**
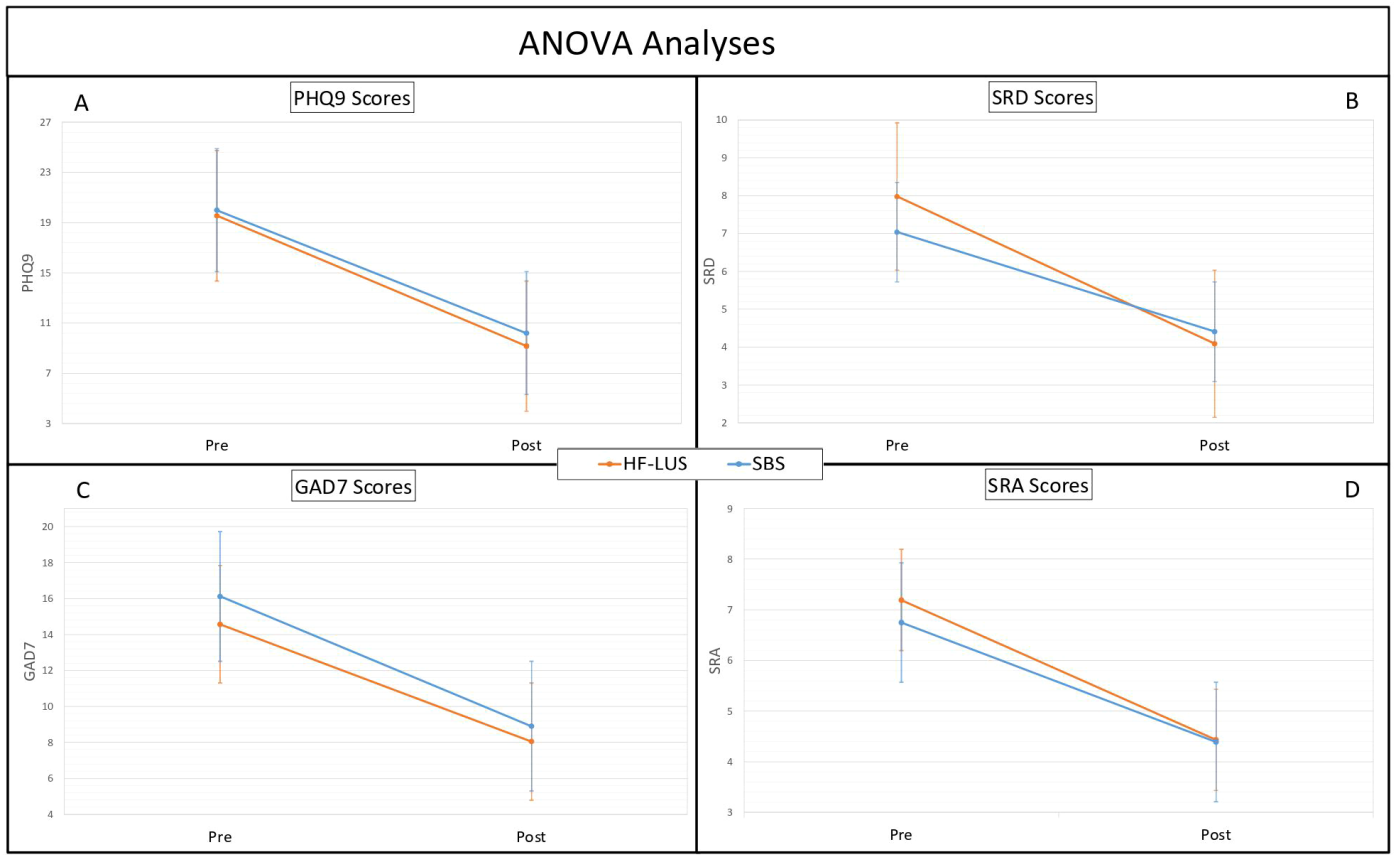
ANOVA analyses. Analysis of Variance (ANOVA) between High-Frequency Left Unilateral Stimulation (HF-LUS) and Sequential Bilateral Stimulation (SBS) cohorts in reported metrics of depression and anxiety. Measures of depression, PHQ9 **(A)** and SRD **(B)**, did not vary significantly between cohorts. Likewise measures of anxiety, GAD7 **(C)** and SRA **(D)**, did not vary significant. Patient Health Questionnaire (PHQ9); Self Reported Depression (SRD); Generalized Anxiety Disorder (GAD7); Self Reported Anxiety (SRA).

**Figure 2:**
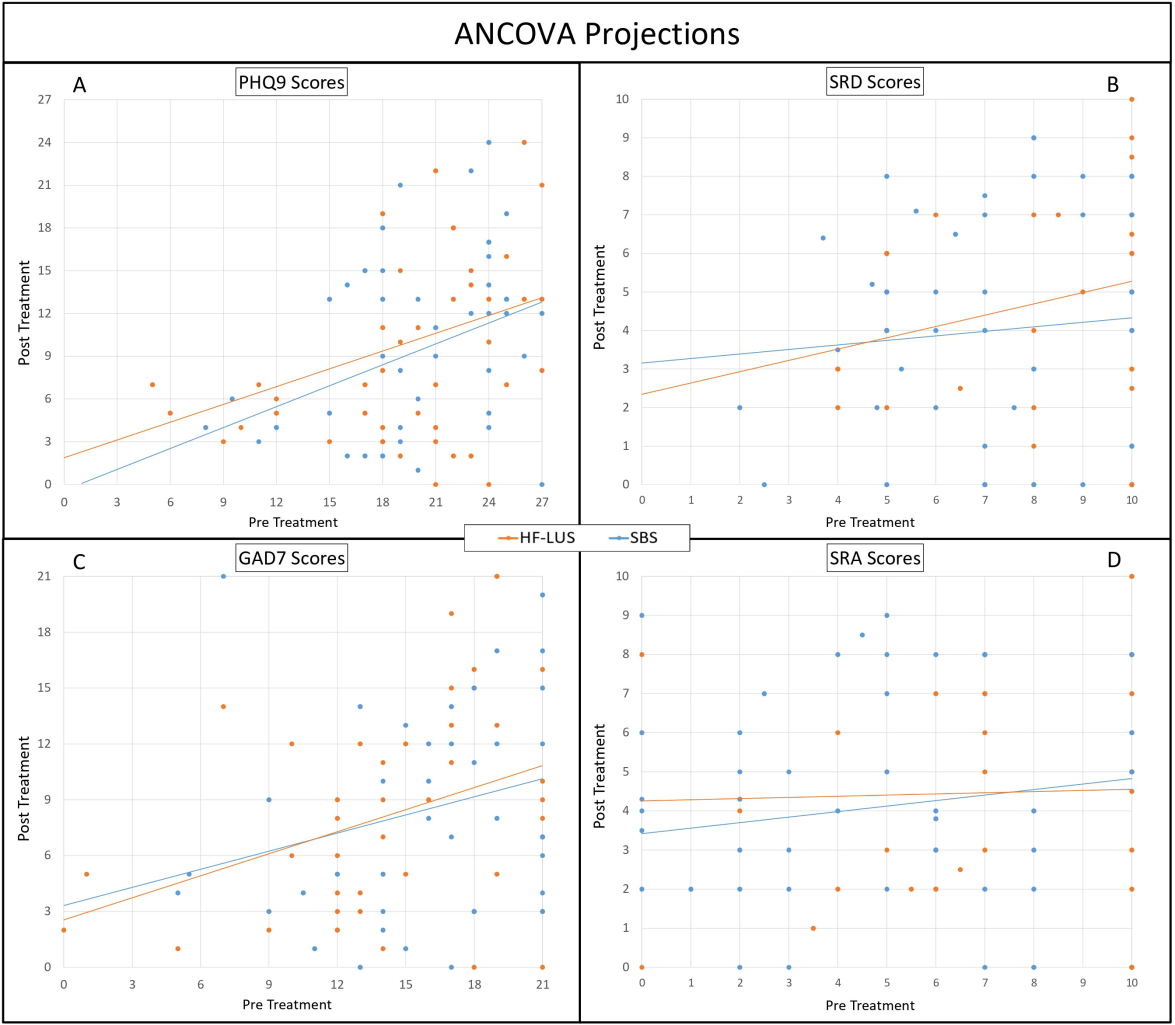
AOVA Projections. Analysis of Covariance (ANCOVA) between High-Frequency Left Unilateral Stimulation (HF-LUS) and Sequential Bilateral Stimulation (SBS) cohorts in reported metrics of depression and anxiety. Trajectories of depression, PHQ9 **(A)** and SRD **(B)**, did not vary significantly between cohorts. Likewise, trajectories of anxiety, GAD7 **(C)** and SRA **(D)**, did not vary significantly. Patient Health Questionnaire (PHQ9); Self Reported Depression (SRD); Generalized Anxiety Disorder (GAD7); Self Reported Anxiety (SRA) 2.

For both the Unilateral and Bilateral Group depression symptom severity significantly improved from pre-post treatment as measured both on the PHQ9 (*F* (1,84) = 210.65, *p* < .001) and SRD (*F* (1,84) = 85.05, *p* < .001). The mean baseline PHQ9 score for the unilateral cohort was 19.55 (SD = 5.48), and for the bilateral cohort was 19.99 (SD = 4.72). Post-treatment, the mean PHQ9 scores decreased to 9.15 (SD = 6.36) and 10.19 (SD = 6.18), respectively, indicating mean improvements of 53.20% and 49.02% (**Figures 1A, B**). There was also a significant effect of time such that the trajectory of scores consistently went down for PHQ9 (*F* (1,628) = 156.73, *p* < .001) and SRD (*F* (1,684) = 80.57, *p* < .001) (**Figures 2A, B**). Consistent with prior findings (39), HF-LUS and SBS did not differ significantly for either the factorial ANOVA nor the ANCOVA analysis indicating that neither the improvement in depression symptoms nor trajectory differed between cohorts.

**Figure 3:**
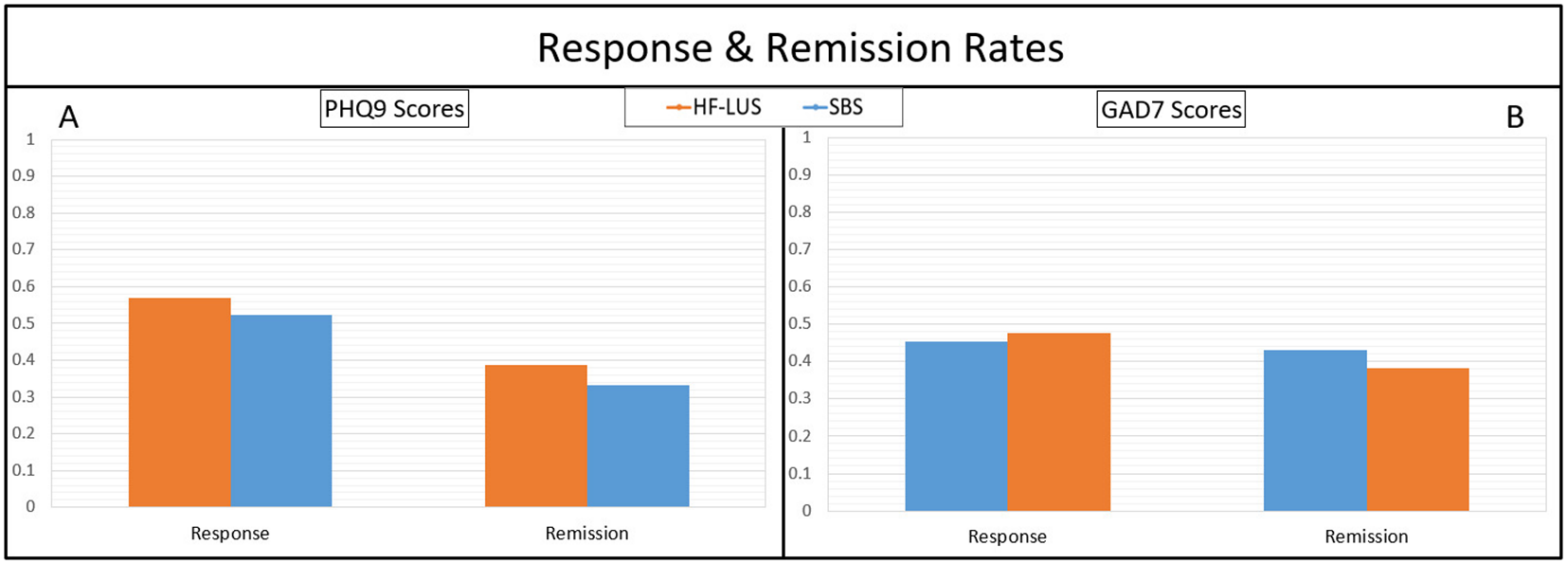
Response rates (≥ 50% improvement with treatment) and remission rates (Post-treatment score ≥5) for measures of depression with PHQ9 **(A)**, and anxiety with GAD7 **(B)**. No significant differences between cohorts were found. Health Questionnaire (PHQ9); Generatlized Anxiety Disorder (GAD7).

Patient responses to treatment with measure of PHQ9 were 56.82% and 52.38%, for the unilateral and bilateral cohorts, respectively. These two percentages were not significantly different. Remission rates of depression were 38.64% and 33.33% for the unilateral and bilateral cohorts, respectively. These two percentages were not significantly different (**Figure 3A**).

### Metrics of anxiety – GAD7 & SRA

For both the Unilateral and Bilateral Group anxiety symptom severity significantly improved from pre-post treatment as measured both on the GAD7 (*F* (1,84) = 94.21, *p* < .001) and SRA (*F* (1,84) = 48.43, *p* < .001). The mean baseline GAD7 score for the unilateral cohort was 14.57 (SD = 5.19), and for the bilateral cohort was 16.12 (SD = 4.57). Post-treatment, the mean GAD7 scores decreased to 8.05 (SD = 5.79) and 8.91 (SD = 6.22), respectively, indicating mean improvements of 44.75% and 44.56% (**Figures 1C, D**). There was also a significant effect of time such that the trajectory of scores consistently went down for GAD7 (*F* (1,612) = 92.55, *p* < .001) and SRA (*F* (1,684) = 282.91, *p* < .001) (**Figures 2C, D**). There was not a significant main effect of cohort nor a cohort by time interaction effect for either the factorial ANOVA nor the ANCOVA analysis indicating that neither the improvement in anxiety symptoms nor trajectory differed between cohorts.

Patient responses to treatment with measure of GAD7 were 45.45% and 47.62%, for the unilateral and bilateral cohorts, respectively. These two percentages were not significantly different. Remission rates of anxiety were 43.18% and 38.10% for the unilateral and bilateral cohorts, respectively. These two percentages were not significantly different (**Figure 3B**).

### Correlation of self-reported anxiety and self-reported depression

**Figure 4:**
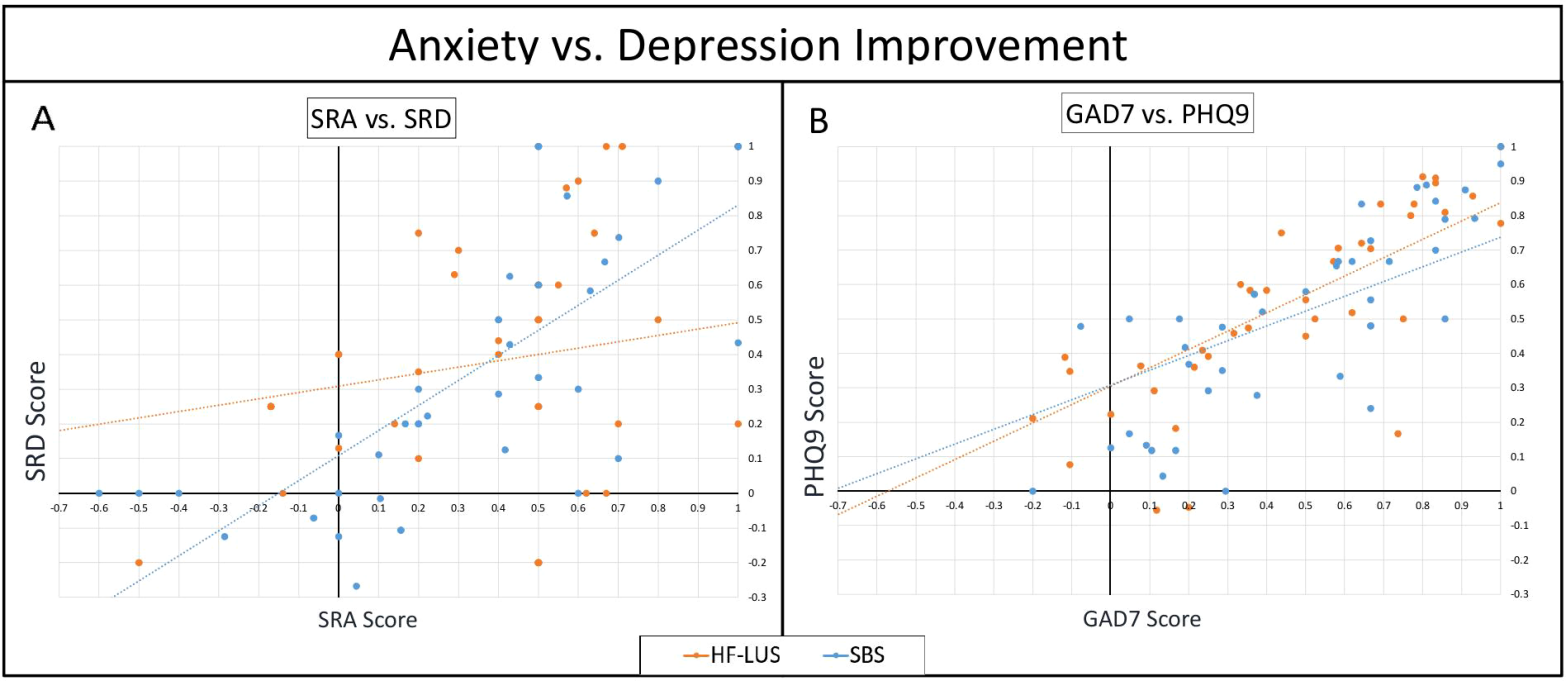
Anxiety vs. Depression Improvement. Scatter plots comparing improvement coefficients (Intake Score - Final score) / Intake score) between measures of anxiety vs. depression. In comparing SRA against SRD scores. **(A)**, while both cohorts had a positive correlation, SBS was significantly higher than HF-LUS. In GAD7 against PHQ9 **(B)** both cohorts had significant positive correlations which were not significantly different. Patient Health Questionnaire (PHQ9); Self Reported Depression (SRD); Generalized Anxiety Disorder (GAD7); Self Reported Anxiety (SRA).

There was a significant positive correlation in the improvements in self-reported anxiety and self-reported depression in both the unilateral and bilateral cohort (Unilateral: r = .397, *p* <.05; Bilateral: r = .721, *p* <.001). Fisher’s transformation (z = 2.19, *p* <.05) confirmed that the correlation was stronger for the SBS cohort as compared to the HF-LUS cohort (**Figure 4A**).

### Correlation of GAD7 and PHQ9

There was a significant positive correlation in the improvements in GAD7 and PHQ9 in both the unilateral and bilateral cohort (Unilateral: r = .768, *p* <.05; Bilateral: r = .738, *p* <.001). Fisher’s transformation confirmed that the correlations were not statistically different (**Figure 4B**).

## Discussion

Following the logic that many traits of anxiety are associated with hyperactivity of the right frontal lobe (19, 32), it is reasonable to consider direct suppression of the right DLPFC with 1 Hz rTMS as a possible adjunctive treatment to HF-LUS for anxious depression, but this is simply not reflected in the data. It may be that the underlying mechanisms leading to anxiety and depression overlap in such a way that HF-LUS is the optimal treatment protocol to address both pathologies simultaneously, leaving stimulation of the right DLPFC of no additional value. If this is the case, our findings have important implications for healthcare systems and resource allocation, as forgoing the redundant right sided stimulation would save time and resources for both patients and clinicians. The SBS protocol requires additional time and resources due to the inclusion of right-sided stimulation, which our data suggest does not confer additional clinical benefit over the unilateral protocol. By adopting the unilateral HF-LUS protocol for patients with anxious depression, clinics and physicians can enhance treatment efficiency, lower costs, and optimize resource utilization without compromising patient outcomes. This approach could lead to increased accessibility of rTMS treatments for a larger patient population. Despite the growing body of evidence supporting rTMS for treatment-resistant depression, its widespread adoption in clinical practice is influenced by factors such as cost, accessibility, and provider training. While the FDA has approved rTMS for anxious depression, its clinical use specifically for anxiety disorders remains off-label. The broader implementation of rTMS for comorbid anxiety conditions may depend on further research, standardization of protocols, and increased insurance coverage to facilitate accessibility. In any case, the lack of appreciable difference in remediation of depressive or anxiety symptoms between these two protocols, which aligns with prior findings (39), leads us to reject our hypothesis. Our results nonetheless serve as reinforcement to the current literature on rTMS. Both treatment protocols had a significant effect on measures of depression and anxiety, further supporting rTMS as an effective modality for treatment resistant MDD, even in the context of anxious comorbidities, as demonstrated by Clark and colleagues (12).

One noteworthy exception to the absence of significant difference was the strong positive correlation in improvement of self-reported scores in the bilateral cohort compared to the relatively weaker positive correlation in the unilateral. While this correlation seems to indicate that self-reported anxiety and depression are improving more uniformly with the SBS protocol, this observation is of little clinical value as improvements in this cohort were not discernably superior to those observed in its counterpart and this pattern was not seen in the standardized PHQ9 and GAD7 scales. Regardless of protocol, our results showed that as depression got better, so did anxiety, or vice versa. While this correlation in anxious depression has already been observed by prior studies (11), further study is warranted to determine the exact mechanism.

### Limitations

This study has several important limitations that need to be acknowledged. First, this is a retrospective, non-randomized study, which inherently introduces biases and confounding factors. One of the major limitations is the lack of random assignment, as patients essentially self-selected their treatment cohorts based on subjective symptom reporting during intake, which could lead to selection bias. Moreover, the open-label nature of the study means both patients and clinicians were unblinded to the treatment protocol, increasing the potential for expectancy effects and bias.

Additionally, the study did not control for medication use, as patients were allowed to continue their psychotropic medication regimens throughout the treatment course. Although no significant differences in medication use between cohorts were observed, this factor could still confound the results. Another limitation is the use of non-validated self-report scales (SRA and SRD) in conjunction with standardized measures like the PHQ-9 and GAD-7. While these scales provided practical real-time assessments, their lack of validation means the accuracy and reliability of these measures may be less robust compared to standardized instruments.

Finally, although we mention the impact of COVID-19, other methodological limitations, such as the lack of control for environmental and situational variables related to the pandemic, may have influenced the results. Future studies should prioritize randomization, blinding, and the use of fully validated measurement tools to reduce potential biases and improve the reliability of findings.

## Conclusion

In conclusion, SBS rTMS for anxious depressive patients may not provide any additional clinical advantages than the FDA cleared HF-LUS rTMS. While both protocols were effective in reducing symptoms of depression and anxiety, forgoing the redundant right sided stimulation would save time and resources for both patients and clinicians.

## Data Availability

The datasets presented in this article are not readily available because the analyses presented in this manuscript are based on a preexisting data set owned by Neuro Wellness TMS Centers Of America. This dataset includes private patient information and is not publicly accessible to maintain confidentiality and adhere to data protection regulations. Requests to access the datasets should be directed to the corresponding author.
